# Statistical learning of a tonal language: the influence of bilingualism and previous linguistic experience

**DOI:** 10.3389/fpsyg.2014.00953

**Published:** 2014-09-03

**Authors:** Tianlin Wang, Jenny R. Saffran

**Affiliations:** Department of Psychology, University of Wisconsin–MadisonMadison, WI, USA

**Keywords:** statistical learning, tonal language, bilingualism, word segmentation, artificial language

## Abstract

While research shows that adults attend to both segmental and suprasegmental regularities in speech, including syllabic transitional probabilities as well as stress and intonational patterns, little is known about how statistical learning operates given input from tonal languages. In the current study, we designed an artificial tone language to address several questions: can adults track regularities in a tonal language? Is learning enhanced by previous exposure to tone-marking languages? Does bilingualism affect learning in this task? To address these questions, we contrasted the performance of English monolingual adults (Experiment 1), Mandarin monolingual and Mandarin–English bilingual adults (Experiment 2), and non-tonal bilingual adults (Experiment 3) in a statistical learning task using an artificial tone language. The pattern of results suggests that while prior exposure to tonal languages did not lead to significant improvements in performance, bilingual experience did enhance learning outcomes. This study represents the first demonstration of statistical learning of an artificial tone language and suggests a complex interplay between prior language experience and subsequent language learning.

## INTRODUCTION

An important component of learning a new language is segmenting words from the speech stream. Such initial learning can be accomplished by using the statistical regularities in fluent speech to determine the boundaries of novel word forms, along with other types of diagnostic cues such as pauses and reliable stress patterns. In speech, sounds that co-occur often are likely to comprise part of a single word, whereas rare sound sequences are likely to mark transitions between words. Both infants and adults are able to track this statistical information and use it to identify novel word forms in an unfamiliar language (e.g., Saffran et al., 1996a,b, 1999; Ludden and Gupta, 2000; Thiessen and Saffran, 2003; Newport and Aslin, 2004). Moreover, sensitivity to the statistical regularity between syllables (*transitional probability*, TP) is not only manifested at the segmental level (i.e., vowels and consonants), but is also evident at the suprasegmental level for both the linguistic and musical domains (Saffran et al., 1999; Saffran, 2003b; Creel et al., 2006; Thiessen and Saffran, 2007; Schön et al., 2008; Hay and Saffran, 2012).

Though research has shown that people can track statistical regularities of syllabic contrasts, prior studies have not investigated languages that rely on tones as integral aspects of lexical representations. Tonal languages are estimated to comprise 60–70% of the world’s languages (Yip, 2002). In syllable–tone languages, pitch variations function in a phonemic manner to distinguish lexical meanings at the syllabic level; these languages therefore employ lexical tones or pitch variations to denote different meanings at the suprasegmental level (e.g., Yip, 2002; Burnham and Mattock, 2007). These pitch contrasts occur regardless of their syntactic or morphological status. In the case of Mandarin Chinese, depending on the pitch contours, the four citation tones in Mandarin can be categorized as either high-level, low-rising, low-dipping, or high-falling – a syllable /pa/ can mean “eight,” “to pull,” “to hold,” or “dad” when carrying these respective tones. Pitch variations are therefore linguistically meaningful in syllable tonal languages because they determine the semantic meaning of a syllable. However, it is currently unclear how cues to word boundaries are weighted in languages that utilize both segmental and suprasegmental information.

There is a general sense that the spoken sound of a tonal language is markedly different from non-tonal languages. Even if a sentence in a non-tonal language is sung, it is not likely to approximate the variations of pitch and tonal contours over individual syllables that are typical of tonal languages. A possible explanation for this phenomenon is that while a sung syllable typically occurs on a single pitch rather than a continuous pitch contour, a tonal syllable includes information about both pitch height (fundamental frequency, or F0) and pitch contour, which can take on either level, rising, falling, or dipping shapes. Adult learners can track regularities between pure tones (Saffran et al., 1999; Saffran, 2003a) as well as sung sequences where a pure tone is super-imposed on a syllable (Schön et al., 2008). In tonal languages like Mandarin, however, pitch variations are used contrastively for lexical meaning and are truly foreign acoustic cues to the ears of non-tonal speakers (Peabody and Seneff, 2009).

To date, the stimuli used in statistical language learning studies have been based on the phonotactics of Indo-European languages, and have not incorporated the linguistic properties of lexical tones. In the current set of experiments, we designed an artificial tone language that resembles syllable–tone languages such as Mandarin and Cantonese in order to examine the process of word segmentation in a tonal context. By utilizing a linguistic cue that differs significantly from Indo-European phonological structure, the artificial tone language simulates a tonal language by providing linguistic regularities at both the suprasegmental and the segmental level. This design also provides an informative test case for assessing adults’ statistical learning ability in processing languages that are typologically different from Indo-European languages. Researchers have intentionally manipulated suprasegmental information in their word segmentation tasks, including stress, intonation, and musical tones (Johnson and Jusczyk, 2001; Thiessen and Saffran, 2003, 2007; Schön et al., 2008). Moreover, learners employ language-specific segmentation strategies that are dependent upon the prosodic organization of a particular language (e.g., Nazzi et al., 2006; Shukla et al., 2007; see Nazzi et al., 2014 for a discussion), such that their prior language knowledge impacts subsequent statistical learning (Finn and Hudson Kam, 2008; Shukla et al., 2011; Lew-Williams and Saffran, 2012). Though such results suggest that there will be differences between tonal and non-tonal speakers in a word segmentation task, the role of lexical tones – a key property that is present in over two-thirds of the worlds’ languages – has not been investigated in prior studies.

While English speakers are able to successfully track statistical properties of languages that range from syllabic artificial languages to natural Italian (Saffran et al., 1996a; Pelucchi et al., 2009), a significant amount of language experience may be necessary before they are able to segment tonal speech. Therefore, the current studies were designed to determine whether prior experience with lexical tones is necessary for adults to segment a tonal language, or whether other non-tonal linguistic experience could also facilitate tonal statistical learning. To begin to address these questions, Experiment 1 examined monolingual English-speaking participants’ performance in a tone–language statistical learning task. Crucially, the materials were created such that the syllable-level statistics and the tonal-level statistics both provided strong and redundant cues to word boundaries. As they utilized stimuli bearing limited similarity to the features of tonal cues, previous studies have shown that the extraction of linguistic information is enhanced when stress patterns coincide with word boundaries (Myers et al., 1996) and also when a speech is sung (Schön et al., 2008), thus suggesting a facilitating role of redundant suprasegmental information. In addition to being a key characteristic of tonal languages, incorporation of lexical tones in experiment stimuli promises to offer further insights into the influence of redundant segmentation cues in statistical learning.

If English monolinguals can make use of the syllable-level statistics and/or the tonal-level statistics, they should succeed at the task. However, if the presence of the unfamiliar tonal structure distracts learners from detecting the syllable-level structure, these materials may be more difficult for English speakers to acquire than the materials used in prior statistical language learning tasks.

## EXPERIMENT 1

In Experiment 1, monolingual English adults participated in a statistical learning task, where they were exposed to an artificial tonal language followed by a forced-choice test (e.g., Saffran et al., 1996a). Compared to prior studies, the artificial language was relatively simple, containing only three trisyllabic words, with two redundant cues to word boundaries (syllable-level statistics and tonal-level statistics). Participants were then tested using a forced-choice design contrasting words versus non-words – sequences of syllable/tone pairs that were reordered relative to the exposure language. Importantly, both types of test items – words and non-words – maintained the trained correspondences between individual syllables and tones. The differences between the test items lay in their sequential statistics at both the syllable and tonal level. The question of interest was whether participants would learn enough about the structure of the artificial tonal language to successfully distinguish between test words and non-words. The speech stream provided identical regularities at both the syllabic and the tonal tiers. Thus, learners could track the syllables alone, the tonal regularities alone, or the two together, as in tonal languages. Given that syllable regularities in the absence of tones are readily acquired by English-learning adults (e.g., Saffran et al., 1996a), we expected that our participants would successfully acquire the artificial tonal language.

### METHOD

#### Participants

Twenty-four English monolingual students at a Midwest university with self-reported normal hearing participated in the experiment. All participants in Experiment 1 and the subsequent experiments provided informed consent in accordance with the University IRB. Participants received extra credit in a psychology course in which they were enrolled. Data from two additional participants were excluded from the analysis due to experimenter error (1) or failure to follow directions (1).

#### Materials

The artificial language consisted of two tiers: syllables and tones. From the material used in the language created by Saffran et al. (1996a), we chose nine syllables (*ta*, *tu*, *ti*, *da*, *du*, *di*, *ba*, *bi*, *gu*) to incorporate into our design. For the tonal tier, three tonal contours (rising, level, falling) were paired with three F0 starting points (register; high, middle, low), resulting in nine tones in total (e.g., high rising, middle falling, low level). To construct the nine tones employed in this language, we surveyed tonal languages that use tones at the syllabic level to form contrastive lexical meaning. In these tonal languages, the span of F0 was suggested to be 87–308 Hz in the case of female speakers (Connell, 2002; Keating and Kuo, 2012), and 10–100 Hz for an individual tone (Lee et al., 2006). Using these distributions, we specified nine tones (see **Table 1**). Using the starting and ending points of F0, we synthesized nine pure tones using the Mbrola speech synthesizer (tcts.fpms.ac.be/synthesis/mbrola.html). The three tonal contours and the three tonal registers we used in our stimuli are present in natural tone languages. The stimuli were recorded by a female native English speaker who does not speak a tonal language. She has perfect pitch and music performance training. She was asked to listen to the synthesized pure tones and “sing” out the same tones with the nine syllables. The recording was conducted one tonal syllable at a time. All tonal syllables were further edited in Adobe Audition to be matched in length (500 ms) and amplitude, while preserving their original pitches.

**Table 1:** Range of F0 (Hz) for all nine tones.

Register height	High		Middle		Low	
Contour shape	Start	End	Start	End	Start	End
Rising	250	330	200	250	150	200
Level	250	250	200	200	150	150
Falling	250	200	200	150	150	50

*Considering that the relation between perception of tones and sound frequencies is not linear, the spread of frequencies for the three registers are 130 Hz (H), 100 Hz (M), and 150 Hz (L). Two native tonal speakers show good discriminatory ability in response to the tonal stimuli.*

To control for arbitrary listening preferences during testing, two counterbalanced conditions of the language were constructed; the non-words in condition A were words in condition B and vice versa. For condition A, the aforementioned syllables and tones were then uniquely paired with one another to create three trisyllabic words: *tadugu*, *bidatu*, *tibadi*. As such, the speech stream consisted of three trisyllabic tonal words, and the words can be uniquely described by either their syllables or tones (see **Table 2**). Each word occurred 30 times, and never repeated twice in succession. Transitional probabilities from the syllabic and tonal tiers thus offered identical and redundant cues to word boundary: 0.5 between words and 1.0 within each word.

**Table 2:** Words and non-words in condition A and B.

		Words			Non-words		
Condition A	Tone	HR MF HF	ML LF LR	MR HL LL	HF MF HR	LR LF ML	LL HL MR
	Syllable	*ta du gu*	*bi da tu*	*ti ba di*	*gu du ta*	*tu da bi*	*di ba ti*
Condition B	Tone	HF MF HR	LR LF ML	LL HL MR	HR MF HF	ML LF LR	MR HL LL
	Syllable	*gu du ta*	*tu da bi*	*di ba ti*	*ta du gu*	*bi da tu*	*ti ba di*

*Registers: H, high; M, middle; L, low. Contours: R, rising; L, level; F, falling.*

For the test items, the non-words were constructed by reversing the order of syllables in each word (e.g., a word whose syllable order was “ti–ba–di” would be rearranged to produce a non-word “di–ba–ti”), resulting in a within-word syllabic/tonal TP = 0. The three non-words in condition A were therefore *guduta*, *tudabi, dibati*. The tone/syllable pairings presented during training were maintained in the test materials. For instance the syllable *ta* is always paired with the high rising tone in words and non-words. Each word was paired exhaustively with each non-word, resulting in 18 test trials.

The syllables were concatenated together into a stream, with 10 ms of silence between syllables, using Adobe Audition. There was no coarticulation between syllables, unlike previous studies of word segmentation. No additional acoustic cues were inserted at word boundaries. The stream was presented 13 times during familiarization, with 390 presentations of each word for a total duration of 9 min.

#### Procedure

Participants were instructed that they would be listening to a non-sense language. They were informed that there were patterns in this language and that their task was to pay as much attention to the language as possible. We included these instructions based on prior results suggesting that adult performance in statistical learning tasks is enhanced by explicit instructions to attend to the stimuli (e.g., Saffran et al., 1997; Turk-Browne et al., 2010).

Participants were assigned to one of the two counterbalanced language conditions: condition A or condition B. After 9 min of listening during the familiarization phase, participants were tested using a forced-choice task between words from the language and non-words. In each test trial, participants heard two trisyllabic strings (one word and one non-word) separated by 500 ms of silence. At the end of each trial, participants were asked to indicate which of the two strings sounded more familiar. The order of presentation of 18 test trials was randomized for each participant. After the test phase, participants filled out questionnaires concerning their language and musical background.

### RESULTS AND DISCUSSION

**FIGURE 1:**
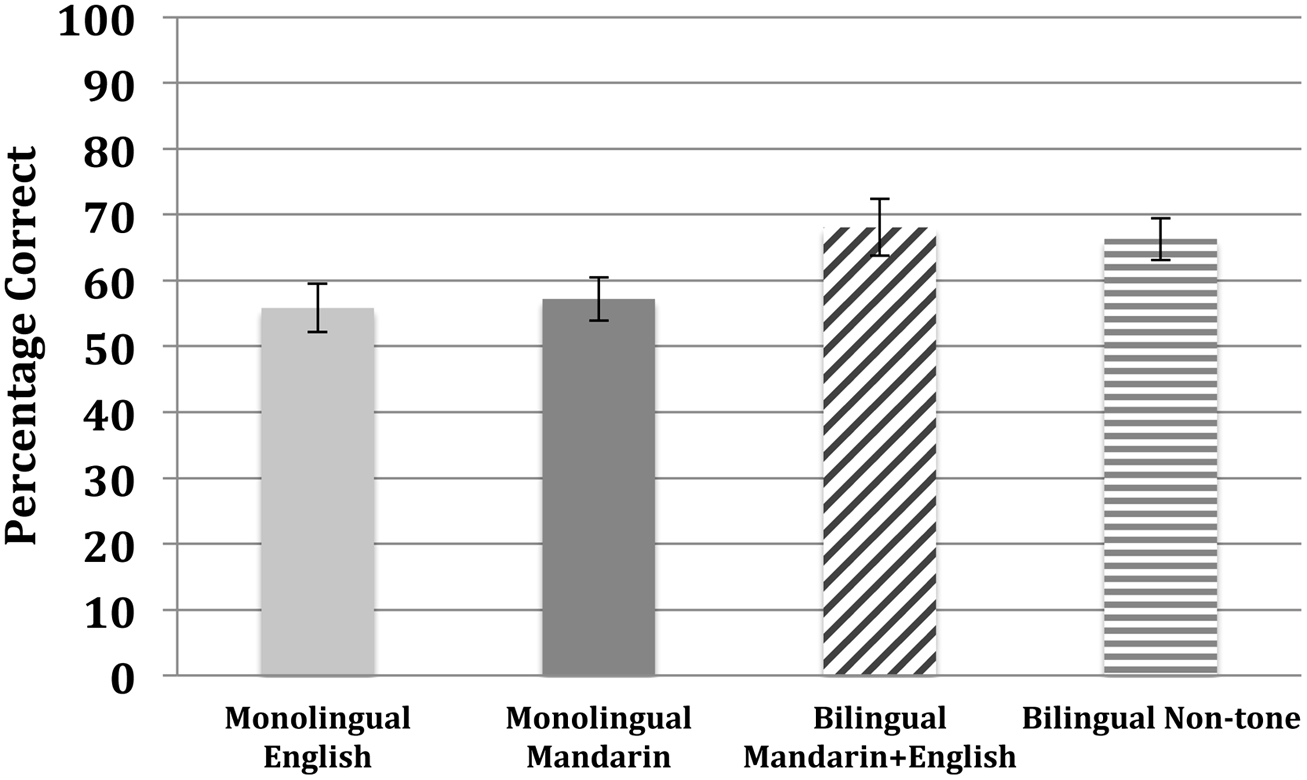
**Average accuracy rate of all four groups.** This figure illustrates the average percentage correct of the four language groups: in Experiment 1, English monolinguals; in Experiment 2, Mandarin monolinguals and Mandarin–English bilinguals; in Experiment 3, non-tonal bilinguals.

We first compared the two counterbalanced familiarization conditions. A *t*-test (all *t*-tests reported are two-tailed) comparing the accuracy rates from the two counterbalanced languages revealed no significant differences [*t*(22) = 1.61, *p* = 0.122], suggesting that there were no *a priori* listening preferences for any of the test words. The two conditions were therefore combined in the subsequent analyses. A one-sample *t*-test showed that the English monolinguals did not perform better than chance (50%) on the forced-choice test [*t*(23) = 1.42, *p* = 0.169], with an average accuracy of 0.55 (SE = 0.03). These participants failed to learn the sequential statistical structure of the tonal artificial language (see **Figure 1**). There was no correlation between participants’ performance and self-reported musical background in Experiment 1 [*r*(22) = 0.11, *p* = 0.601].

Given that there is ample research showing that adults can regularly track either segmental or suprasegmental cues in statistical learning tasks (Saffran et al., 1996a; Saffran, 2003b; Schön et al., 2008), we had expected this tonal artificial language to be relatively easy to learn – it offers two sets of redundant and equally informative cues (i.e., tonal and syllabic). The failure of the English monolinguals to discern the statistical properties of this language was surprising, and suggests that they were unable to utilize either of the redundant cues to word boundaries available in these materials.

The results of Experiment 1 suggest that there may be attributes of these materials that made the task markedly more challenging than previous statistical learning tasks. One possible factor that could have given rise to our participants’ difficulty in performing this task is the acoustically prominent nature of lexical tones. Lexical tones are carried by the fundamental frequency in speech. Though vowel quality and coarticulation may slightly condition the realization of tones, lexical tones nevertheless exist at the suprasegmental level, i.e., a tone’s pitch contour and pitch height can be consistently realized irrespective of segmental characteristics of the syllable with which they are paired (Liberman and Pierrehumbert, 1984; Shen, 1992; Cao, 2002; Liu et al., 2007). In contrast to lexical stress, which can also change a word’s meaning (e.g., REcord versus reCORD), lexical tone alters meaning in a far more dramatic fashion and usually results in semantically unrelated lexical items (e.g., /pa/ can mean “eight,” “to pull,” “target,” or “father” depending on the four tones in Mandarin). Such lexical contrast cannot be accomplished by any suprasegmental properties inherent in Indo-European languages. Adult second language learners with non-tonal linguistic backgrounds frequently report that tones are the hardest aspect of a tonal language to acquire (Peabody and Seneff, 2009), suggesting an inherent complexity of tones as perceived by non-tonal language speakers. Furthermore, in a study looking at tonal discrimination abilities across languages, native Mandarin speakers outperformed non-tonal participants in a task requiring them to discriminate between Thai tones (Wayland and Guion, 2004). That result suggests that experience with lexical tones in one language facilitates the discrimination of tones in another language, demonstrating the important role of prior linguistic experience in processing lexical tones.

Therefore, we hypothesized that previous exposure to a tonal language might facilitate learning in our tonal statistical learning task. To address this hypothesis and examine the potential effect of a more variable language experience on word segmentation in a new language, we next examined the performance of native Mandarin speakers from the same university community as the participants from Experiment 1. These participants were bilingual in Mandarin and English. While they attended the same university as the monolingual English-speaking participants from Experiment 1, these participants differ two ways: exposure to Mandarin and bilingual status. To control for the latter factor, we also collected data from a group of monolingual college students in Mainland China. This group came from a different country and university setting than the participants in Experiment 1, but share their monolingual status. We thus included two groups of Mandarin-speaking participants in Experiment 2, bilingual international students in the USA and monolingual students in Mainland China.

## EXPERIMENT 2

As native speakers of Mandarin, a lexical tone language, both the Mandarin monolinguals and Mandarin–English bilinguals tested in Experiment 2 are intimately familiar with the properties of tones. If the failure of monolingual English speakers in Experiment 1 was due to the aural interference produced by lexical tones, both groups of Mandarin speakers should be able to succeed in the segmentation task. This experiment also afforded the opportunity to examine the effects of more variable language experiences (bilingual versus monolingual) on a challenging statistical learning task.

### METHOD

#### Participants

Twenty-four monolingual Mandarin speakers and 24 Mandarin–English bilinguals participated in this experiment. The Mandarin–English bilinguals were recruited from the same Midwestern University as the participants in Experiment 1, and received extra credit in a general psychology course in which they were enrolled. The Mandarin monolinguals were recruited from a university in Mainland China and received monetary compensation. From the language questionnaire filled out by participants, all bilingual Mandarin–English participants reported that they spoke Mandarin at home, were fluent in English, and used English for both academic and social purposes on a daily basis. Though the monolinguals tested in Mainland China reported English exposure for an average of 3–5 h/week from seventh to 12th grade, they also reported having minimal exposure to English during the most recent 2 years of their college career. None of the Mandarin monolinguals reported functional usage of English. Data from six additional participants were excluded from the analysis because of experimenter error (2), participant termination of the experiment (2), participant falling asleep (1), and participant-reported hearing loss (1).

#### Materials and procedure

The materials and procedure were identical to those used in Experiment 1.

### RESULTS AND DISCUSSION

To examine whether tonal experience facilitates word segmentation in processing this speech stream, we compared the accuracy rate of both groups (shown in **Figure 1**) to chance performance using single-sample *t*-tests (all *t*-tests two-tailed; effect sizes reported for *t*-tests are Cohen’s *d*). The Mandarin monolinguals successfully discriminated words from non-words as shown by performance significantly better than chance [mean = 0.57, SE = 0.03; *t*(23) = 2.18, *p* < 0.05, *d* = 3.30]. The Mandarin–English bilinguals also performed significantly better than chance [mean = 0.68, SE = 0.04; *t*(23) = 4.28, *p* < 0.001, *d* = 6.36]. There was no correlation between participants’ performance and self-reported musical background for the monolingual Mandarin group [*r*(22) = 0.10, *p* = 0.650] or the Mandarin–English bilingual group [*r*(22) = -0.05, *p* = 0.828]. While the Mandarin monolinguals performed better than chance (unlike the English monolinguals in Experiment 1), a *t*-test comparing the Mandarin and English monolingual groups suggests that experience with lexical tones did not facilitate the detection of structure in the tonal artificial language [*t*(46) = 0.49, *p* = 0.626].

In order to explore the potential effect of more variable language experience on word segmentation in a new language, we used an independent samples *t*-test to compare the performance of two Mandarin-speaking groups. The Mandarin–English bilinguals scored significantly higher than the Mandarin monolinguals [*t*(46) = 2.12, *p* < 0.05, *d* = 3.11], suggesting that familiarity with both Mandarin and English resulted in better learning than familiarity with Mandarin alone.

These results suggest that being bilingual in Mandarin and English improves performance in tracking statistical regularities in a tonal language-learning environment. One possible explanation for these results lies in the phonological structure of the two languages. Spoken Mandarin words are predominantly disyllabic (Chu and Qian, 2001), while spoken English words frequently range from mono- to pentasyllabic (see CELEX; Baayen et al., 1995; Denes, 2005). Compared to the Mandarin monolinguals, the Mandarin–English bilinguals’ superior performance on this task may be due, at least in part, to their experience with multi-syllabic English words that require tracking of within-word TPs for more than two syllables. Since Mandarin speech is disyllabic, the monolingual Mandarin participants might have found the chunking of trisyllabic units challenging in the current task. Indeed, previous research suggests that at least for infants, expectations regarding word length affect statistical learning: learners expecting trisyllabic words find it difficult to detect disyllabic words, and vice versa (Lew-Williams and Saffran, 2012). If the performance of the Mandarin monolinguals is indeed affected by their prior experience with word length, the bilinguals might be better prepared to segment the trisyllabic units from this artificial language due to their familiarity with tracking units of this length.

An alternative, though not necessarily contradictory, explanation is that there are benefits associated with being bilingual that are unrelated to the specific languages being acquired. Experience with multiple languages is associated with improved learning of words’ form-meaning links (Cenoz and Valencia, 1994; Sanz, 2000; Cenoz, 2003; Keshavarz and Astaneh, 2004; Kaushanskaya and Marian, 2009), and bilinguals outperform monolinguals in implicit learning tasks (Klein, 1995; Kovács and Mehler, 2009). Bilingual learning advantages (e.g., *phonological working memory*, Papagno and Vallar, 1995; Service et al., 2002; Majerus et al., 2008; Adesope et al., 2010; *inhibitory control*, Bialystok, 1999; Bialystok et al., 2004; Costa et al., 2008; *implicit learning*, Bartolotti et al., 2011) may affect the processing of novel speech.

While previous research has shown that bilinguals possess a better phonological working memory (Service et al., 2002; Majerus et al., 2008; Adesope et al., 2010), and that an enhanced capacity for the statistical learning of word forms is correlated with a well-developed phonological working memory (Misyak and Christiansen, 2007), it is still unclear how bilinguals will perform when encountering a novel linguistic cue that is unrepresented in either of their already-known languages. Thus, in Experiment 3, we tested a new group of bilingual adults who had no prior exposure to tonal languages in order to determine whether the learning advantage observed in Experiment 2 for the Mandarin–English group was due to the specific languages these participants speak or to more general factors associated with bilingualism.

## EXPERIMENT 3

In order to tease apart the role of bilingualism and prior linguistic experience, we added a non-tonal bilingual (e.g., two non-tonal languages such as English and Spanish) group in Experiment 3 to create a 2 (tonal experience) × 2 (bilingualism) factorial design across the three experiments. If prior linguistic experience with lexical tonality is required in this task, we should observe a main effect of tonal experience, such that that the tonal groups outperform the non-tonal groups. If advantages associated with bilingualism enhance performance on this task, we should observe a main effect of bilingualism, such that the non-tonal bilinguals tested in Experiment 3 would also be able to succeed in the word segmentation task (despite their lack of tonal language experience). The inclusion of the non-tonal bilingual group in Experiment 3 also affords an opportunity to compare monolingual and bilingual participants from the same university, none of whom speak a tonal language.

### METHOD

#### Participants

Fifteen college-aged non-tonal bilinguals (e.g., two non-tonal languages such as English and Spanish) participated in the experiment. All participants were recruited from the same university as in Experiments 1 and 2 (bilingual group) and reported normal hearing. Participants received extra credit in a general psychology course in which they were enrolled. Participants were prescreened for bilingualism in a mass survey, and reported in the language background questionnaire that they spoke a non-tonal language other than English at home, or that they know another language fluently or natively. The non-tonal languages included Spanish, Korean, German, Polish, and French (see **Table 3** for details).

**Table 3:** Language and musical background of four groups of participants.

	English monolinguals	Mandarin monolinguals	Mandarin–English bilinguals	Non-tonal bilinguals
L2 proficiency (scale 1–7)	3.17 (0.14)	2.33 (0.18)	5.63 (0.15)	6.07 (0.12)
L2 frequency of use (%)	19.17 (1.46)	13.33 (1.97)	55.42 (1.59)	34.67 (3.89)
Musical training (years)	5.35 (0.61)	1.25 (0.44)	4.17 (0.59)	3.93 (0.71)

*Means and SE (in parentheses). L2 spoken by the non-tonal bilinguals include the following: Spanish (6), English (4), Korean (1), French (1), Polish (1), German (1), Japanese (1). Other than the 4 English-as-an L2 speakers (whose L1 was Korean), all other non-tonal bilinguals’ L1 was English. Although some Gyeongsang Korean dialects are considered tonal, most Korean dialects as well as standard Korean are not. None of the Korean speakers spoke the tonal dialects. All monolingual Chinese subjects are from the Mandarin dialect group.*

#### Materials and procedure

The materials and procedure were identical to those used in Experiment 1.

### RESULTS AND DISCUSSION

As shown in **Figure 1**, the non-tonal bilinguals performed significantly better than chance [mean = 0.66, SD = 0.12; *t*(14) = 5.13, *p* < 0.01, *d* = 1.89]. Comparing the results from the English monolingual group from Experiment 1 and the non-tonal bilinguals from Experiment 3 to the two Mandarin-speaking groups from Experiment 2, the main effect of Tonal Experience was not significant [*F*(1,83) = 0.68, *p* = 0.41]. However, a comparison between the two bilingual groups (Mandarin–English bilinguals from Experiment 2 and non-tone bilinguals from Experiment 3) and the two monolingual groups (English monolinguals from Experiment 1 and Mandarin monolinguals from Experiment 2) reveals that the main effect of Bilingualism was significant [*F*(1,83) = 9.52, *p* < 0.05, *d* = 0.64]; the bilingual participants outperformed the monolingual participants. There was also an interaction among the groups [*F*(3,83) = 3.258, *p* < 0.05, *η*^2^ = 0.105]. Independent samples *t*-tests with Bonferroni correction showed that the non-tone bilinguals’ accuracy rate on the word segmentation task was higher than their monolingual English-speaking peers [*t*(37) = 2.28, *p* < 0.025, *d* = 1.20], but not higher than the monolingual Mandarin group [*t*(37) = 1.87, *p* = 0.07] or the Mandarin–English bilinguals [*t*(37) = 0.3 *p* = 0.76]. This pattern of results suggests that while being bilingual improves performance in this task, it does not lead to better performance than does cue-specific linguistic knowledge acquired from prior experience with tonal languages. There was no correlation between participants’ performance and self-reported musical background for the non-tonal bilingual group [*r*(13) = 0.34, *p* = 0.22].

## GENERAL DISCUSSION

In this series of experiments, we tested four groups of participants from varying linguistic backgrounds in a tonal statistical language-learning task. Without any prior experience with tones, an English monolingual group failed to perform above chance (Experiment 1), suggesting a difficulty in processing these two redundant statistical cues. To further examine the prominence of tonal cues, we then tested two groups of Mandarin speakers: Mandarin monolinguals and Mandarin–English bilinguals (Experiment 2). Both tonal language groups succeeded in discriminating words from non-words, though tonal experience did not lead to statistically better performance in the task relative to the English learners from Experiment 1. Additionally, the bilingual Mandarin–English speakers outperformed their Mandarin monolingual peers, as well as the English monolinguals. Given previous findings regarding the advantages associated with being bilingual, we then tested a non-tonal bilingual group in order to tease apart the influence of bilingualism versus cue-specific linguistic experience on the ability to extract regularities from this tonal artificial speech stream (Experiment 3). Results from the non-tonal bilinguals suggest that bilingualism alone does facilitate statistical learning in this task.

The present study allows us to investigate the separate and combined influences of bilingualism and prior linguistic experience (here, with tonal languages) on statistical learning. While previous research suggests that bilingualism facilitates learning another language (Cenoz and Valencia, 1994; Sanz, 2000; Cenoz, 2003; Keshavarz and Astaneh, 2004; Kaushanskaya and Marian, 2009), in the current study we found that the specific types of linguistic cues present in previous bilingual experience also play a prominent role in one’s ability to track regularities in a new linguistic environment.

Mandarin–English bilinguals in Experiment 2 were the most successful group that we tested. While the Mandarin monolinguals tested in Experiment 2 had little experience with words longer than two syllables, and the English monolinguals tested in Experiment 1 had no prior experience with lexical tones, the Mandarin–English bilinguals were highly familiar with both tonal cues as well as a wider span of possible word lengths due to their daily usage of English. The fact that this group outperformed the two monolingual groups suggests that previous linguistic experiences matching the characteristics of the new language (here, word length) may improve learning outcomes. Bidelman et al. (2013) suggest that speakers of tone languages could outperform speakers of non-tonal languages in a series of cognitive tasks, implying that a tonal linguistic background may have also contributed to the Mandarin–English bilinguals’ superior performance.

However, there was no significant difference between the performance of the non-tonal bilinguals tested in Experiment 3 and the Mandarin–English bilinguals tested in Experiment 2, suggesting another possible explanation for our pattern of results: there are benefits associated with being bilingual that facilitated learning in this task. Comparing the non-tonal bilinguals to the English monolinguals, we see that even though neither group had previous linguistic experience with tonal cues, the non-tonal bilinguals still identified the words more reliably than the English monolinguals. Surprisingly, they performed as well as the Mandarin monolinguals who already had experience navigating tones that are part of their native linguistic repertoire. These results suggest that while experience with particular linguistic cues facilitates learning a new language, bilingualism also contributes to a comparatively successful learning outcome. Thus, even though the monolingual Mandarin speakers and the non-tonal bilingual group performed the task with similar rates of success, the current study does not address whether or not they employed the same cue-weighing strategies or whether they paid attention to the same set of cues in this statistical learning task.

The ability to learn novel forms in a new sequence or pattern of information via statistical regularities may be indirectly improved by previous bilingual experience. General advantages associated with bilingualism in this context have long been postulated. Bilinguals may develop a more effective implicit learning mechanism than monolinguals as the result of acquiring the words and grammar of multiple languages (Nation and McLaughlin, 1986). This increased efficiency may also contribute to bilinguals’ improved incidental learning of word forms while listening to speech.

Another consequence of bilingualism for cognition is an improved phonological working memory (Service et al., 2002; Majerus et al., 2008; Adesope et al., 2010) that results from acquiring and processing a large vocabulary that extends across multiple languages. An enhanced capacity for the statistical learning of word forms is correlated with a well-developed phonological working memory (Misyak and Christiansen, 2007), suggesting that phonological working memory can be used to retain large chunks of speech in memory long enough for the transitions between syllables to be compared. In addition, working memory may help to update the relative frequency of different syllabic transitions. Based on statistical patterns in speech, likely word candidates can be identified and transferred from working memory to long-term memory. According to this view, bilinguals should thus be expected to outperform monolinguals in the statistical learning of word forms in a novel language due to the development of a superior phonological working memory.

Additionally, bilingualism has also been shown to confer better inhibitory control (Bialystok, 1999; Bialystok et al., 2004; Costa et al., 2008). Considering the fact that our tonal artificial language provided strong redundant statistical cues (i.e., tonal and syllabic), a small lexicon of just three words, and a very straightforward test contrast between words and non-words, it was surprising that English monolinguals showed no evidence of learning. It is possible that the non-tonal bilinguals may possess a greater degree of flexibility: they may either be able to pay attention to both tonal and syllabic cues or be able to exhibit better inhibitory control and therefore not be distracted by the novel tonal cues. Either of these two possible alternatives could explain the link between their performance and executive functions.

Relatedly, research has also shown that the listener’s language experience affects later statistical learning (e.g., Shukla et al., 2007, 2011), and may also result in different cue-weighting strategies (Toro et al., 2009; Tyler and Cutler, 2009). Therefore, the identical statistical regularities provided by the tonal and the syllabic tiers may be treated differentially by monolinguals versus bilinguals. For the English monolinguals in particular, it is possible that rather than functioning as redundant cues and thus facilitating word segmentation, the tonal and syllabic information interfered with each other, resulting in a “more is less” learning situation.

To summarize, our findings suggest that there are advantages conferred by bilingualism in the learning outcome of novel word forms. Though there also seems to be a likelihood that confluence between previous linguistic experience (here, word length) and the characteristics of the new language can facilitate such learning as well, the originally hypothesized role of tonal experience was not supported by the results. To date, there has been much attention paid to the link between bilingual experience and executive control (e.g., inhibitory control, task switching, task monitoring, response suppression), whereas little discussion has focused on how specific elements of previous linguistic experience may interact with features of a new language – at least during the initial stages of the learning process. By employing an artificial language designed to approximate a specific set of linguistic features, the results suggest that both familiarity with specific forms of linguistic cues and bilingual experience can affect success in word-form learning. When prior linguistic experience fails to provide learners expectations concerning the cues particular to the language to be learned, bilingualism can facilitate learning in this word segmentation task. There are, however, significant limitations associated with utilizing an artificial language. Though prior artificial language findings have been subsequently confirmed with natural language studies (e.g., Pelucchi et al., 2009), ecological validity is always a concern in artificial designs. Future work is needed to not only investigate and isolate the specific mechanisms of bilingualism that underlie this facilitating effect, but also validate these hypotheses and results while employing natural tonal materials. It will also be of great interest to manipulate the test materials to determine whether the successful learners relied more heavily on the tonal-level statistics, the syllable-level statistics, or the confluence between the two. Examining the role of inhibitory control as well as phonological working memory in this context will also be essential for a more complete understanding the role of bilingual experience.

In conclusion, learning about statistical regularities in speech is not just about tracking whatever cues happen to be available. The present study demonstrates the contribution of the specific linguistic experience with which learners arrive at the lab; the results are also consistent with the findings of earlier studies that point towards learning advantages associated with bilingualism. This study presents the first demonstration of statistical learning of an artificial tone language and suggests that there is a complex interplay between language experience and language learning, raising exciting possibilities for future research.

## Conflict of Interest Statement

The Guest Associate Editor Margarita Kaushanskaya declares that, despite being affiliated to the same institution as authors Tianlin Wang and Jenny R. Saffran, the review process was handled objectively and no conflict of interest exists. The authors declare that the research was conducted in the absence of any commercial or financial relationships that could be construed as a potential conflict of interest.
